# Three-layer model for the control of epidemic infection over multiple social networks

**DOI:** 10.1007/s42452-023-05373-0

**Published:** 2023-04-29

**Authors:** Ali Nasir

**Affiliations:** 1 Control and Instrumentation Engineering Department, King Fahd University of Petroleum and Minerals, Dhahran, Saudi Arabia; 2 Interdisciplinary Research Center for Intelligent Manufacturing and Robotics, King Fahd University of Petroleum and Minerals, Dhahran, Saudi Arabia

**Keywords:** Markov decision process, Epidemics control, Two-layer networks, Markov chain approach

## Abstract

**Abstract:**

This paper presents a hierarchical approach for controlling the spread of an epidemic disease. The approach consists of a three-layer architecture where a set of two-layer multiple social networks is governed by a (third) top-layer consisting of an optimal control policy. Each of the two-layer social networks is modeled by a microscopic Markov chain. On top of all the two-layer networks is an optimal control policy that has been developed by using an underlying Markov Decision Process (MDP) model. Mathematical models pertaining to the top-level MDP as well as two-layer microscopic Markov chains have been presented. Practical implementation methodology using the proposed models has also been discussed along with a numerical example. The results in the numerical example illustrate the control of an epidemic using the optimal policy. Directions for further research and characterization of the optimal policy have also been discussed with the help of the same numerical example.

**Article Highlights:**

- An optimal approach for controlling the spread of an epidemic infection.
- The approach is able to model the uncertainties involved in the problem.
- The approach is able to cater for the underlying social network.

## Introduction

Recent outbreak of coronavirus has reminded us to be more vigilant in developing effective mathematical models for epidemic control. Mathematical models for epidemics serve two main purposes. First is to predict the dynamics of a disease and second is to devise appropriate control measures to prevent the spread of the same. There has been a lot of research on developing various mathematical models for epidemics. Consequently, there are multiple existing models (mostly based on the discrete-time difference equations or continuous-time differential equations) such as SIR (susceptible-infected-recovered) model [1], and SEIR (susceptible-exposed-infected-recovered) model [2]. Recently proposed models employ network topologies into the development of epidemics spread and control models [3]. Some stochastic models have also been proposed, but there are very limited attempts on applying stochastic control for epidemic control [3].

Latest research on epidemic control has considerable focus on reducing the rate of contact among the individuals in a social setup [4] but the downside of reduced social activities has also been highlighted [5], hence a balance is required between controlling the spread of an epidemic and avoiding new social issues in doing so. In principle, there are three main classes of epidemic models [6], i.e., differential equations based models [7], agent-based (networked) models [8], and Markov decision process (MDP) based models [9, 10]. Each type of model has its own advantages and drawbacks. For example, the differential equations based models are flexible and offer insights such as equilibrium points and stability analysis. On the other hand, MDP based models offer handling of uncertainty and calculation of optimal decision making policy. Finally, the graph-based or agent based or networked models offer a bottom-up approach where the agents can be heterogeneous representing various population groups based on age, gender, social status etc. One can also perform sensitivity analysis on such models for identification of influential parameters.

The concept of having multiple layers in an epidemic model has previously been introduced in [11] where two-layer networked model is proposed with one layer comprising of SIR-type agents and the other layer comprising of aware-unaware agents. This model has recently been discussed further in [12] where the effectiveness of the model has been demonstrated and a mathematical result has been derived regarding the threshold of epidemic spread. Furthermore, in [8], it has been investigated how positive and negative preventive information affects the spread of the epidemic.

Although the two-layer model discussed in [11] and [12] does present an effective way of modeling the epidemics, there still exist two problems. One issue is scalability because the population of a town or city may be as much as hundreds of thousands. The second issue is the incorporation of uncertainty into the model that arises from variations in the interaction level among various subgroups of the population. In-fact there is no clear partition among various social groups in the models existing in the literature. For example, schools, colleges, universities, shopping malls, parks, offices, railway stations, airports etc. Represent various platforms for social interaction and incorporation of these platforms into the epidemic models can provide a better insight into predicting and controlling of an epidemic. The focus of this paper is to reduce the drawbacks and increase the advantages of an epidemic model by combining different approaches into a single multi-layer epidemics model.

**Fig. 1:**
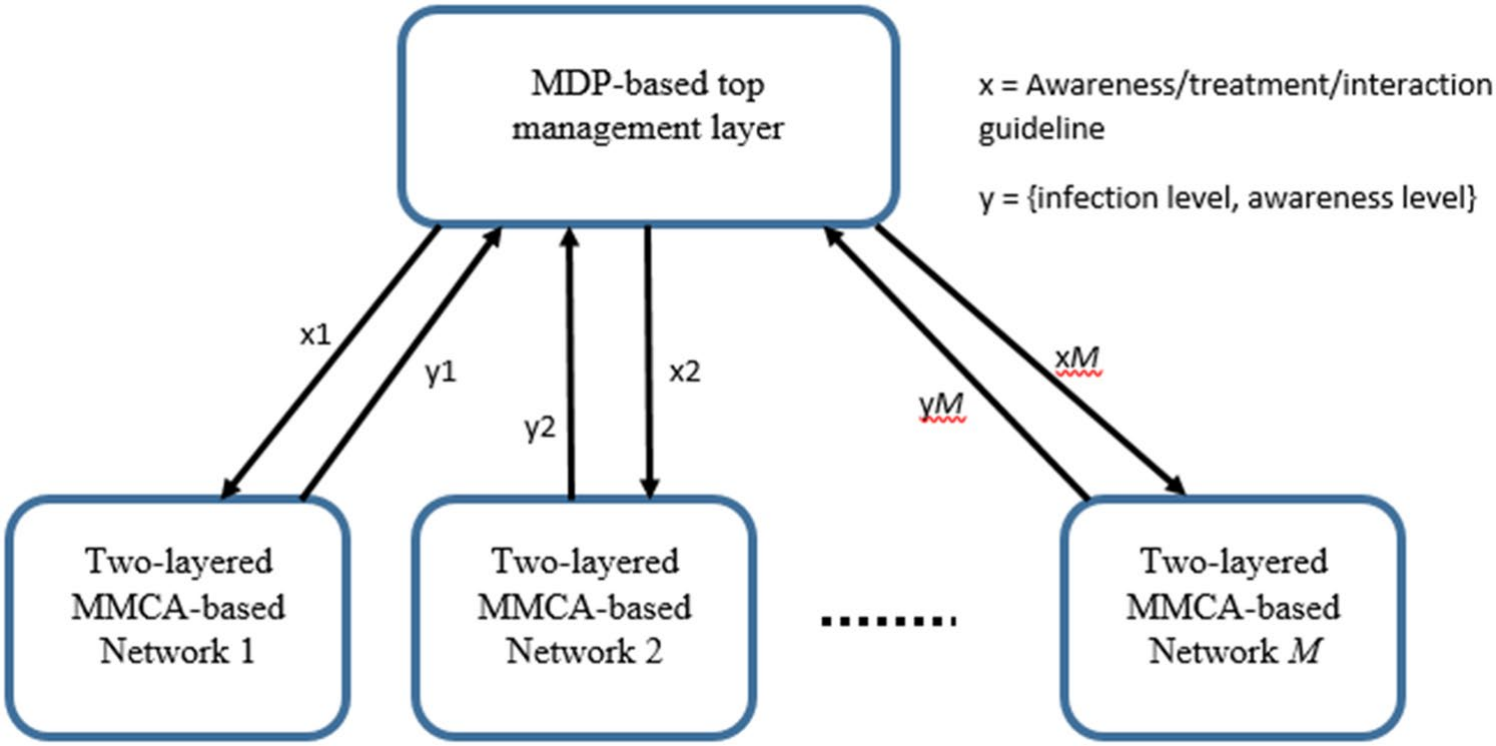
Multi-layered model framework

The main idea of this paper is to propose a three-layer model where the top layer manages an awareness level, treatment, and interaction among various subgroups in the bottom layer. The bottom layer consists of multiple two-layer networks where each network represents a certain social interaction platform, e.g., a school, an office, a household, a shopping mall, a cinema etc. Each two-layer network is based on a microscopic Markov chain approach (MMCA) as discussed in [8] and [12] with some modifications needed to incorporate the difference among various social platforms. Figure 1 shows the framework of the proposed model. As indicated in the figure, the top layer uses an MDP-based model for generating optimal control policy regarding the treatment, awareness, and social interaction guidelines for the bottom layer networks. The bottom layer networks convey the infection level (i.e., fraction of the population within the network that has been infected) to the top layer along with awareness level. The top layer uses information from each of the networks in the bottom layer to decide the next set of decisions according to a pre-calculated optimal policy. Some closely relevant research contributions include the dynamic programing based optimal policy for immunization [13, 14] and work on an awareness layer within the social networks [15, 16]. Another interesting dimension of related work involves modeling of human behavior and prediction of the risk of epidemic spread [17, 18]. For example, in [17], the authors have proposed two different models for human behavior, i.e., the information forgetting curve (IFC) model and the memory reception fading and cumulating (MRFC) model. It has been shown that MRFC is well suited for modeling the epidemics that are more lethal whereas IFC is suitable for low-risk epidemics. Both of the models are based on two-layer networks.

Major reason for proposing the third layer in this paper is that the two-layer network-based approach (although it does provide great insights into the spread of the disease) does not provide any guidance regarding how to optimally control the spread of the disease. Controlling the spread is by no means a straightforward task because it requires the information regarding the available resources, the cost associated with the consumption of the resources, the effectiveness of the resource utilization policy etc. Therefore, a third layer is necessary for efficiently controlling the spread of the disease under various situations. The third layer from Fig. 1 is able to cater for the uncertainties involved in the problem and is able to enable the calculation of a decision policy that is optimal with respect to the available resources and the known structure of the uncertainties involved in the problem.

The use of artificial intelligence in epidemic control is endorsed by recent work on the epidemics control spread prediction using machine learning [19]. In this regard, there is another relevant approach based on game theory [20]. Both of these approaches have close link with an MDP-based approach such as the one in this paper. However, in the existing approaches, the link to the social and contact models is insufficient. Also, MDP has the advantage over other artificial intelligence-based approaches that it can model uncertainties in the problem. In terms of social lock downs and limitations on the social interactions, there have been multiple studies. For example, approaches to lift the lockdown in London [21], the impact of human mobility in China [22], the impact of travel restrictions on the spread of epidemics [23] etc. Further studies focus on the impact of the heterogenous nature of the population of the vaccination policies [24], the spread of the disease despite the restrictions on the social gatherings [25], and sideways contact tracing in large gatherings [26]. All of the recent work endorses the utilization of a complex decision-making approach that involves modeling of the social and contact layers and that utilizes multiple strategies, i.e., a combination of quarantine, vaccination, and treatment. Therefore, the focus of the approach of the current study is along the same lines.

The major contribution of the current work is to propose a multi-network model of the society with a centralized control layer to minimize the spread of an epidemic disease. Modeling the society as comprising of multiple networks (as proposed) allows for incorporation of real-life social interaction platforms in the society. The centralized control over all the networks represents the local governing bodies that are responsible for providing the treatment facilities, social awareness, and the standard operating procedures to minimize the spread of an epidemic disease. Practical implementation of the proposed model and the calculation of the associated control policy requires the identification of different social interaction platforms in the society and some initial statistical data regarding the spread of the disease. Furthermore, the costs of treatment and awareness campaigns are also needed for implementation of the proposed approach. Once the prerequisite information is provided for, our proposed model can be used to calculate the optimal policy that provides the best decision (whether to launch an awareness campaign, vaccinate people, or launch a treatment campaign) based on the given situation (number of people infected, susceptible, aware, unaware, recovered, etc., belonging to various social and contact networks).

The paper is organized such that Section II presents mathematical models, Section III includes the implementation strategy and optimal policy calculation. Section IV shows how to apply the proposed model and understand the optimal policy. Concluding remarks are written in Section V.

## Mathematical model

The proposed mathematical model for epidemics is discussed in this section. We begin with the discussion of bottom layer MMCA-based model that has been adopted from [11, 12] followed by the top layer model that is indigenously developed in this paper.

### A. Two-layer network model based on MMCA

**Fig. 2:**
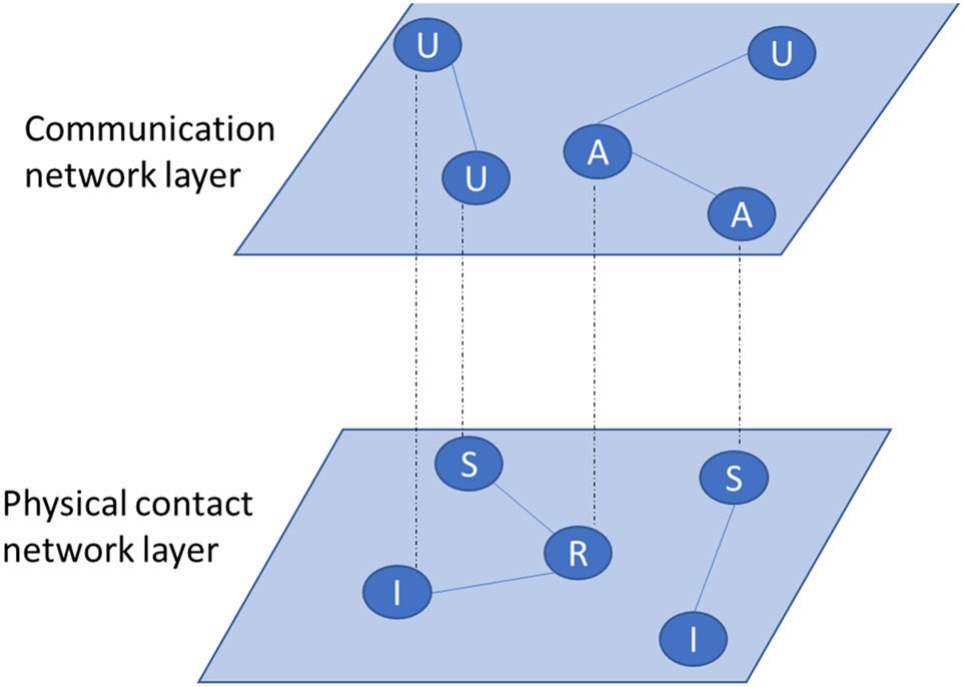
Framework of two-layer multiplex networks. Upper layer indicates communication among the individuals and lower layer indicates physical contact. The dotted line indicates the relationship between the two layers where an aware/unaware individual could be susceptible, infected, or recovered

Each two-layer network in our proposed model consists of a physical contact layer and a virtual communication layer as discussed in [12]. Figure 2 presents the framework of the two-layer network considered here. As shown in the figure, an individual in the communication layer can either be aware (*A*) or unaware (*U*). An individual in the network can become aware with probability $\lambda$ and can forget about gained awareness with probability $\delta$.

In the physical contact layer, an individual can be susceptible (*S*), infected (*I*), or recovered (*R*). Let $\beta$ be the probability with which a susceptible individual can become infected (for our purposes, $\beta$ can be chosen to have different value for different networks/social subgroups, consequently, each network has its own $\beta$). Similarly, consider the curing rate to be $\mu$. The probability of getting an infection for the aware individuals may be reduced by a factor $\gamma \in \left(\mathrm{0,1}\right]$, i.e., ${\beta }^{A}=\gamma {\beta }^{U}=\gamma \beta$ where ${\beta }^{U}=\beta$.

**Fig. 3:**
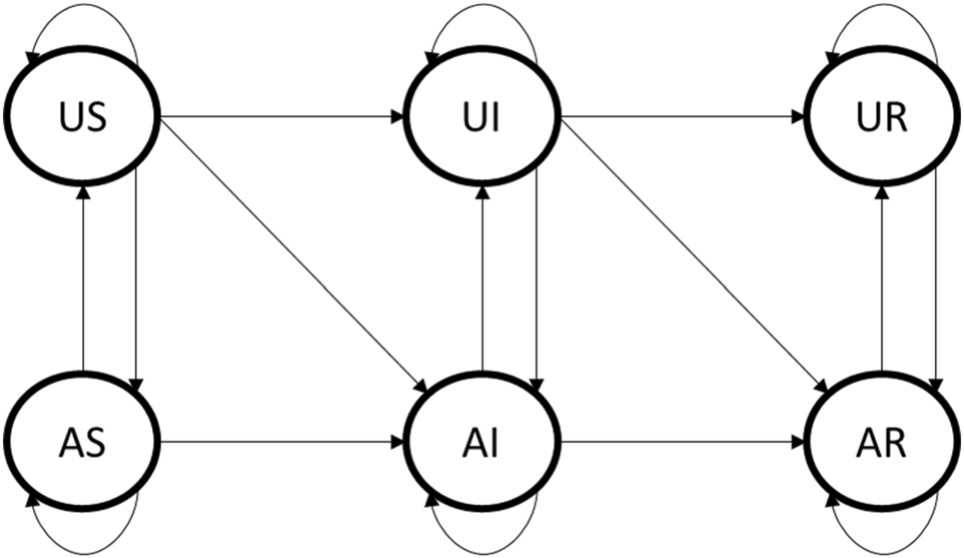
State transitions in a network

With the two layers discussed above, each node within each network has six possible states, i.e., aware and susceptible (*AS*), unaware and susceptible (*US*), aware and infected (*AI*), unaware and infected (*UI*), aware and recovered (*AR*) and unaware and recovered (*UR*). At any given time, *t*, and individual *i* can be in any one of the six possible states. Furthermore, an individual at time step *t* may transition from any (of the six) state to any (of the six) state in the next time step ($t+1$). The allowable state transitions include remaining in the *same* state as well. The corresponding state probabilities are given by ${p}_{i}^{AS}\left(t\right),{p}_{i}^{US}\left(t\right),{p}_{i}^{AI}\left(t\right),{p}_{i}^{UI}\left(t\right),{p}_{i}^{AR}\left(t\right)$ and ${p}_{i}^{UR}(t)$. Note that the subscript $i$ in the state probabilities indicates a node (an individual person) in the two-layer network. Hence ${p}_{i}^{US}(t)$ is the probability for an individual $i$ being unaware and susceptible at time $t$ (similar definitions apply to the other five probabilities). Figure 3 shows the dynamics within a network in the form of possible transitions between the states. For example, an unaware and susceptible individual may become aware and infected or unaware and infected or aware and susceptible or stay unaware and susceptible. Network-related probabilities are used in the next section for deriving the state transition probabilities for the top layer MDP model. The matrices indicating adjacency among the individuals in the contact layer and the communication layer are $A=\left({a}_{ij}\right)$ and $B=({b}_{ij})$ respectively. Where ${a}_{ij}=1$ if two individuals i and j are adjacent in the contact layer and ${a}_{ij}=0$ otherwise. Similarly, ${b}_{ij}=1$ if two individuals i and j are adjacent in the communication layer and ${b}_{ij}=0$ otherwise. Furthermore, in the communication layer, the probability for an individual i to remain unaware at time t is denoted as ${r}_{i}(t)$. On the contact layer, the probabilities of susceptible individuals not getting infected from the neighbors are denoted as ${q}_{i}^{A}(t)$ and ${q}_{i}^{U}(t)$ for aware and unaware individuals respectively. The expressions for these probabilities are as follows

1 $${r}_{i}\left(t\right)=\prod_{j}\left[1-{a}_{ji}\left({p}_{j}^{AI}\left(t\right)+{p}_{j}^{AS}\left(t\right)+{p}_{j}^{AR}\left(t\right)\right)\lambda \right]$$

2 $${q}_{i}^{A}\left(t\right)=\prod_{j}\left[1-{b}_{ji}\left({p}_{j}^{AI}\left(t\right)+{p}_{j}^{UI}\left(t\right)\right){\beta }^{A}\right]$$

3 $${q}_{i}^{U}\left(t\right)=\prod_{j}\left[1-{b}_{ji}\left({p}_{j}^{AI}\left(t\right)+{p}_{j}^{UI}\left(t\right)\right){\beta }^{U}\right]$$

Note that in (1), the probability of remaining unaware for an individual *i* increases if its neighbors (represented by the index $j$) are unaware. Similarly, the probability of not getting infected increases if the neighbors are not infected. The values of the probabilities may vary from network to network, but the equations/relationships remain the same.

The dynamical evolution of the six states in MMCA model using (1), (2), and (3) is presented as follows

4 $$ \begin{gathered} p_{i}^{U} S(t + 1) = p_{i}^{U} S(t)r_{i} (t)q_{i}^{U} (t) + p_{i}^{A} S(t)q_{i}^{U} (t)\delta \hfill \\ p_{i}^{UI} \left( {t + 1} \right) = \left( {1 - \nu } \right)\left[ {\left( {1 - \mu } \right)\left( {p_{i}^{AI} \left( t \right)\delta + p_{i}^{UI} \left( t \right)r_{i} \left( t \right)} \right) + \left( {p_{i}^{AS} \left( t \right)\delta + p_{i}^{US} \left( t \right)r_{i} \left( t \right)} \right)\left[ {1 - q_{i}^{U} \left( t \right)} \right]} \right] \hfill \\ p_{i}^{AS} \left( {t + 1} \right) = \left( {p_{i}^{US} \left( t \right)\left( {1 - r_{i} \left( t \right)} \right) + p_{i}^{AS} \left( t \right)\left( {1 - \delta } \right)} \right)q_{i}^{A} \left( t \right) \hfill \\ p_{i}^{AI} \left( {t + 1} \right) = p_{i}^{AI} \left( t \right)\left[ {\delta \left( {1 - \mu } \right)\nu + \left( {1 - \delta } \right)\left( {1 - \mu } \right)} \right] + p_{i}^{UI} \left( t \right)\left[ {r_{i} \left( t \right)\left( {1 - \mu } \right)\nu + \left( {1 - r_{i} \left( t \right)} \right)\left( {1 - \mu } \right)} \right] + p_{i}^{AS} \left( t \right)\left[ {\delta \left( {1 - q_{i}^{U} \left( t \right)} \right)\nu + \left( {1 - \delta } \right)\left( {1 - q_{i}^{A} \left( t \right)} \right)} \right] + p_{i}^{US} \left( t \right)\left[ {r_{i} \left( t \right)\left( {1 - q_{i}^{U} \left( t \right)} \right)\nu + \left( {1 - r_{i} \left( t \right)} \right)\left( {1 - q_{i}^{A} \left( t \right)} \right)} \right] \hfill \\ p_{i}^{U} R(t + 1) = p_{i}^{U} I(t)r_{i} (t)? + p_{i}^{A} I(t)\delta \mu + p_{i}^{A} R(t)\delta + p_{i}^{U} R(t)r_{i} (t) \hfill \\ p_{i}^{AR} \left( {t + 1} \right) = p_{i}^{UI} \left( t \right)\left( {1 - r_{i} \left( t \right)} \right)\mu + p_{i}^{AI} \left( t \right)\left( {1 - \delta } \right)\mu + p_{i}^{AR} \left( t \right)\left( {1 - \delta } \right) + p_{i}^{UR} \left( t \right)\left( {1 - r_{i} \left( t \right)} \right) \hfill \\ \end{gathered} $$

where $$t+1$$ refers to the next time step (assuming the duration of a time step $$dt=1$$ unit) and $$\nu $$ is self-perception rate which represents the transition probability from the state *UI* to the state *AI*, i.e., self-perception rate determines how quickly the awareness is created among the unaware individuals. Also, note that as $$t\to \infty $$, the probabilities in (4) reach a steady state value, i.e., $${p}_{i}^{**}\left(t+1\right)={p}_{i}^{**}\left(t\right)={p}_{i}^{**}$$. Furthermore, the following equality holds at all times.

5 $${p}_{i}^{US}\left(t\right)+{p}_{i}^{UI}\left(t\right)+{p}_{i}^{AS}\left(t\right)+{p}_{i}^{AI}\left(t\right)+{p}_{i}^{AR}\left(t\right)+{p}_{i}^{UR}\left(t\right)=1$$

It has been shown in [12] that under the assumptions that ${p}_{i}^{AI}+{p}_{i}^{UI}={p}_{i}^{I}={\epsilon }_{i}\ll 1$, ${p}_{i}^{AR}\to 0$ and ${p}_{i}^{UR}\to 0$ (when the initial value of infected nodes is small enough), the threshold of epidemic outbreak is given by

6 $${\beta }_{c}^{U}=\frac{\mu }{{\Lambda }_{max}}$$

where ${\Lambda }_{max}$ is the largest eigen value of matrix *H* with ${h}_{ji}=\left[1-\left(1-\gamma \right){p}_{i}^{A}\right]{b}_{ji}$ and ${p}_{i}^{A}={p}_{i}^{AS}+{p}_{i}^{AI}+{p}_{i}^{AR}$.

### B. Top-layer MDP Model

While the bottom-level networks represent the dynamics of various social subgroups, the top layer MDP model is primarily used for control purposes. An MDP model consists of a set of discrete states (*X*), set of decisions (*D*), and cost function (*J*), a transition probability function (*F*), and a discount factor $\eta \in \left(\mathrm{0,1}\right)$ that determines whether to focus on long term decision making ($\eta \approx 1$) or short-term decision making ($\eta \approx 0$).

States in an MDP model signify the information required (or available) in order to make a decision. In the problem at hand, we have a set of social networks with a population where each individual may be susceptible, infected, or recovered. Consequently, one may argue that we need the information regarding the status of each individual in a network for making a decision regarding the treatment as done in [9] and [10]. But the problem with such an approach is that the computational complexity involved in the calculation of the optimal policy is prohibitive. Therefore, a more innovative approach is required in defining the states of the MDP model. Here we notice that the decisions to be made by the optimal policy (as discussed later) is regarding an awareness campaign, and a treatment drive. An awareness campaign in real life could be a public service message on the television and radio or in the form of billboards on highways or in the downtown area of a city. Similarly, treatment drives (in real life scenario) may be launched in local hospitals for treating the infected individuals. This involves provision of necessary medical equipment and medicine. Based on the decisions to be made, there is information of two types that is needed in a state, i.e., the infection ratio of each network (${z}_{i}$ for ${i}^{th}$ network) and the awareness ratio of each network (${g}_{i}$ for ${i}^{th}$ network). The infection ratio is defined as the ratio of infected individuals to the total population of the network. Consequently, the infection ratio ranges between 0 and 1 where the value 1 means that the whole population of the network is infected. Similarly, the awareness ratio is the ratio of aware individuals in a network to the total population of the network. The value of awareness ratio also ranges between 0 and 1 where the value 1 means that all of the individuals in a network are aware of the epidemic spread. As a result, the state space is represented as

7 $$ \begin{gathered} X = \left\{ {x_{1} ,x_{2} , \ldots ,x_{n} } \right\} \hfill \\ x_{i} = \left\{ {z_{1} ,z_{2} , \ldots ,z_{M} ,g_{1} ,g_{2} , \ldots ,g_{M} } \right\} \hfill \\ z_{j} ,g_{j} \in \left\{ {0,{\Delta },2{\Delta }, \ldots ,1} \right\} \hfill \\ \end{gathered} $$

In (7), the infection level and the awareness level information is required for calculating the desirable control input. Both types of information have been assigned discrete values between 0 and 1 with equal spacing $\Delta$ between any two adjacent values (for a network with $m$ individuals, $\Delta =1/m$). Note that the proposed model is suitable for the SIS (Susceptible-Infected-Susceptible) type of situations where an individual may get re-infected after the recovery from disease. For situations where some of the population is immune (recovered) to the disease, the information of infection ratio alone will still be useful, but we would be ignoring the fact that not all who are not infected are susceptible and hence the decision regarding the awareness campaign may be suboptimal.

As discussed earlier, the set of decisions includes launching an awareness campaign and launching a treatment drive. In our model, a hospital (or set of hospitals) can be regarded as a social subgroup (represented by a two-layered network as described in the previous subsection). A treatment drive executed on a network shall remove infected individuals from that network and add the same to the network representing hospitals. Consequently, the decision of executing treatment drive can only be implemented on $M-1$ networks and not on the network representing hospitals. On the other hand, an awareness campaign can be executed in all networks including hospitals since we are allowing for the possibility of unaware infected individuals. Mathematically, the set of decisions is written as

8 $$D=\left\{{d}_{1}^{A},{d}_{2}^{A},\dots ,{d}_{M-1}^{A},{d}_{M}^{A}{,d}_{1}^{T},{d}_{2}^{T},\dots ,{d}_{M-1}^{T},{d}_{0}\right\}$$

All of the above decisions are Boolean variables with default value *false*. Executing a decision means turning the corresponding decision variable value to be equal to *true* for a single decision epoch (a decision epoch is the time interval between two consecutive decisions and is specified by the user). For example, ${d}_{0}$ is a Boolean no-decision option that is included to enable the control policy to do nothing (switching ${d}_{0}=true$ means no decision regarding awareness or treatment for one decision epoch, ${d}_{0}=false$ by default). This decision is useful in the situations where the treatment or awareness campaigns are not needed or are too expensive. Also, ${d}_{1}^{A}$ refers to the decision of launching an awareness campaign in network 1 and so on. Launching an awareness campaign (${d}_{j}^{A}$) increases the probability of rise in the awareness ration (${g}_{j}$) of the corresponding (${j}^{th}$) network. Similarly, ${d}_{1}^{T}$ refers to the decision of launching a treatment campaign in network 1 and so on (except the network $M$ which is assumed to be the hospital where all of the individuals are already under treatment). Notice that although we have assumed only one hospital, one may assume more hospitals in the community. Moreover, launching a treatment campaign (${d}_{j}^{T}$) increases the probability of fall in the infection ration (${z}_{j}$) of the corresponding (${j}^{th}$) network.

State transition probabilities are of two types. One is the probability of increase or decrease in awareness ratio (${g}_{j}$) in *j*^*th*^ network. Recall that the probability for an individual *i* in a network to remain unaware is ${r}_{i}$ and the probability of forgetting is $\delta$ (independent of the network topology). To simplify the mathematics, it has been assumed that the maximum number of individuals in each network (*m*) is the same and that $\Delta$ is the inverse of the number of individuals in a network. Finally, it has been assumed that during a single time step, ${g}_{j}$ can either remain same, increase by $\Delta$ or reduce by $\Delta$. This means that awareness and unawareness spread at the rate of one individual at a time (recall that an individual in the network can become aware with probability $\lambda$ and can forget about gained awareness with probability $\delta$). Increasing this rate would require the mathematical relation for the transition in ${g}_{j}$ to accommodate more possibilities which can be done but is avoided here. Before discussing the transition probabilities of the MDP layer, it is important to mention that the state probabilities for the lower layer networks have steady states [12], e.g., the steady state value of ${p}_{i}^{UI}\left(t\right)$ is $p_{i}^{UI} : = \mathop {\lim }\limits_{t \to \infty } p_{i}^{UI} \left( t \right)$. Also, since we assume multiple networks (each with $m$ nodes), we define the individual node state probabilities as ${p}_{i,j}^{AS}$ for the steady state probability of aware and susceptible node $i$ of network $j$ (note that since Eqs. (1–6) are for a single network, therefore, additional subscript $j$ is not needed there). Consequently, the transition probability expression for ${g}_{j}$ under above assumptions is given as

9 $$P\left({g}_{j}\left(t+1\right)={g}_{j}\left(t\right)\right)=\overline{{p }_{j}^{A}}+\overline{{p }_{j}^{U}}-2\overline{{p }_{j}^{A}}.\overline{{p }_{j}^{U}}$$

10 $$P\left({g}_{j}\left(t+1\right)={g}_{j}\left(t\right)+\Delta \right)=\overline{{p }_{j}^{A}}\left(1-\overline{{p }_{j}^{U}}\right)$$

11 $$P\left({g}_{j}\left(t+1\right)={g}_{j}\left(t\right)-\Delta \right)=\overline{{p }_{j}^{U}}\left(1-\overline{{p }_{j}^{A}}\right)$$

where $\overline{{p }_{j}^{A}}$ and $\overline{{p }_{j}^{U}}$ for *j*^th^ network are given by,

12 $$\overline{{p }_{j}^{A}}=\frac{1}{m}\sum_{i=1}^{m}\left({p}_{i,j}^{AS}+{p}_{i,j}^{AR}+{p}_{i,j}^{AI}\right)$$

13 $$\overline{{p }_{j}^{U}}=\frac{1}{m}\sum_{i=1}^{m}\left({p}_{i,j}^{US}+{p}_{i,j}^{UR}+{p}_{i,j}^{UI}\right)$$

Here, (9) gives the probability that the fraction of awareness in each subnetwork *j* remains same. Expression (10) provides the probability of an increase in awareness and (11) presents the probability of decrease in awareness. Note that the sum of right-hand sides in (9), (10), and (11) is 1. Furthermore, the sum of right-hand side of Eqs. (12) and (13) is also 1 (this is by chance since all of the six states are involved in the two equations. We shall see later in Eqs. (17) and (18) that this is not the case). The equations in (10) are based on average likelihood of someone becoming aware from being unaware and no one becoming unaware from being aware whereas, Eq. (11) is based on average likelihood of someone becoming unaware from being aware and no one becoming aware from being unaware in the network $j$. Note that executing an awareness campaign in the network *j* would be modeled by reduction in ${r}_{i}$ (please refer to Eq. (1)) for each individual in the network by a factor $\tau \in \left(\mathrm{0,1}\right)$ that would be the inverse of the strength of the awareness campaign, i.e., stronger the awareness campaign, lower the value of $\tau$. Please note that the terms on the right-hand side of Eqs. (12) and (13) are the steady state values of the network state probabilities in Eqs. (1–6). The difference between $t$ and $t+1$ (in Eqs. (9–11)) is the time duration between two consecutive MDP states (note that this is different from the time step used in the Eqs. (1–6) because for each network, we use steady state probabilities in the MDP model). This duration is equal to one decision epoch. The exact value of the duration of the decision epoch is chosen by the user and the calculation formulas for the probabilities are not affected by this choice. Usually, the duration of an epoch may be a day, or a week or a value in between depending upon the severity of the situation and the rate of spread of the disease. Finally, note that the right-hand sides of probabilities in (9, 10, 11) and (14, 15, 16) are independent of $t$, this means that the MDP state transition probabilities are stationary in nature. This is important for calculating the optimal policy for infinite horizon decision-making.

The second type of probability in the MDP model is the infection ratio probability. In this, recall that the recovery rate of an individual in a network is $\mu$ and the infection rate is $\beta$. Consequently, under the assumptions stated earlier, the transition probability for the infection ratio of *j*^th^ group (${z}_{j}$) is given by

14 $$P\left({z}_{j}\left(t+1\right)={z}_{j}\left(t\right)\right)=\overline{{p }_{j}^{I}}+\overline{{p }_{j}^{R}}-2\overline{{p }_{j}^{I}}.\overline{{p }_{j}^{R}}$$

15 $$P\left({z}_{j}\left(t+1\right)={z}_{j}\left(t\right)+\Delta \right)=\overline{{p }_{j}^{I}}\left(1-\overline{{p }_{j}^{R}}\right)$$

16 $$P\left({z}_{j}\left(t+1\right)={z}_{j}\left(t\right)-\Delta \right)=\overline{{p }_{j}^{R}}\left(1-\overline{{p }_{j}^{I}}\right)$$

where $\overline{{p }_{j}^{I}}$ and $\overline{{p }_{j}^{R}}$ for *j*^th^ network are given by,

17 $$\overline{{p }_{j}^{I}}=\frac{1}{m}\sum_{i=1}^{m}\left({p}_{i,j}^{AI}+{p}_{i,j}^{UI}\right)$$

18 $$\overline{{p }_{j}^{R}}=\frac{1}{m}\sum_{i=1}^{m}\left({p}_{i,j}^{UR}+{p}_{i,j}^{AR}\right)$$

Here, (14) gives the probability that the fraction of infection in each subnetwork *j* remains same. Expression (15) provides the probability of an increase in infection fraction and (16) presents the probability of decrease in infection. Note that the sum of right hand sides in (14), (15), and (16) is 1. Note that executing a treatment campaign in a network results in replacement of an infected individual with a recovered individual which increases the right hand side of (18) and reduces the right hand side of (17) depending upon the strength of treatment campaign.

It is important here to mention how the assumption of a fixed maximum number of individuals shall work especially when the treatment campaign is supposed to bring individuals into the hospital network. The proposed model works by replacing empty beds in the hospitals with recovered individuals, and every time an individual is shifted from another network *j* to a hospital, an imaginary recovered individual is placed in the network *j* to keep the total number of individuals consistent. Also, this implies that if the hospital network has 100% infected individuals, the treatment decision cannot be executed which is true in real life situations.

Based on the above discussion, the state transition probabilities can be specified using the joint probability model as follows

19 $$ F\left( {x\left( {t + 1} \right),x\left( t \right),d\left( t \right)} \right) = P\left( {g_{1} \left( {t + 1} \right), \ldots ,g_{M} \left( {t + 1} \right),z_{1} \left( {t + 1 } \right), \ldots ,z_{M} \left( {t + 1} \right)|g_{1} \left( t \right), \ldots ,g_{M} \left( t \right),z_{1} \left( t \right), \ldots ,z_{M} \left( t \right),d\left( t \right)} \right) $$

Finally, the cost function involves cost of being in ‘bad’ states (i.e., the states with low awareness ratio and/or high infection ratio) and the cost of executing an awareness campaign as well as the cost of a treatment drive. A mathematical expression for the cost function is as follows

20 $$ J\left( {x\left( t \right),d\left( t \right)} \right) = \mathop \sum \limits_{j = 1}^{M} \left( {\alpha_{1} z_{j} + \alpha_{2} \left( {1 - g_{j} } \right)} \right)k_{j} + \alpha_{3} \left( {d\left( t \right) \in D_{A} } \right) + \alpha_{4} \left( {d\left( t \right) \in D_{T} } \right) $$

where,

21 $${D}_{A}=\left\{{d}_{1}^{A},{d}_{2}^{A},\dots ,{d}_{M-1}^{A},{d}_{M}^{A}\right\}, {D}_{T}=\left\{{d}_{1}^{T},{d}_{2}^{T},\dots ,{d}_{M-1}^{T}\right\}$$

In (20), ${\alpha }_{i},i\in \left\{\mathrm{1,2},\mathrm{3,4}\right\}$ are positive constants representing the cost associated with the decisions and the states. For example, ${\alpha }_{1}$ represents the cost associated with the infection ratio within a network, ${\alpha }_{2}$ represents the cost associated with the lack of awareness, and ${\alpha }_{3}$ represents the cost associated with the awareness campaign. Also, the priority levels (${k}_{j},j\in \left\{\mathrm{1,2},\dots ,M\right\}$) are constants that are used to differentiate the cost associated with sensitive population network (e.g., pregnant women, children, and elderly) and resilient population network (e.g., healthy youngsters). Note that the only decision ${d}_{0}$ is without any cost. Also, the cost in a state is associated with accumulative infection ratio (${z}_{j}$) and awareness ratio (${g}_{j}$) scaled by the priority level (${k}_{j}$).

Finally, the selection of discount factor $\eta$ in this case may be chosen to have any values close to one if it is desirable to have the resulting optimal control policy be able to consider long-term effects. For short-term results, value of $\eta$ can be selected close to zero.

## Calculation of control policy and practical implementation

In this section, the integration of the MMCA based models and the top layer of MDP model is discussed followed by a possible approach for calculation of the optimal policy.

### Integration of MMCA based models with MDP

Working of the multi-layered approach has been sketched earlier in Fig. 1. Here we provide the details of the communication between the MMCA based networks and the MDP-based model.

**Fig. 4:**
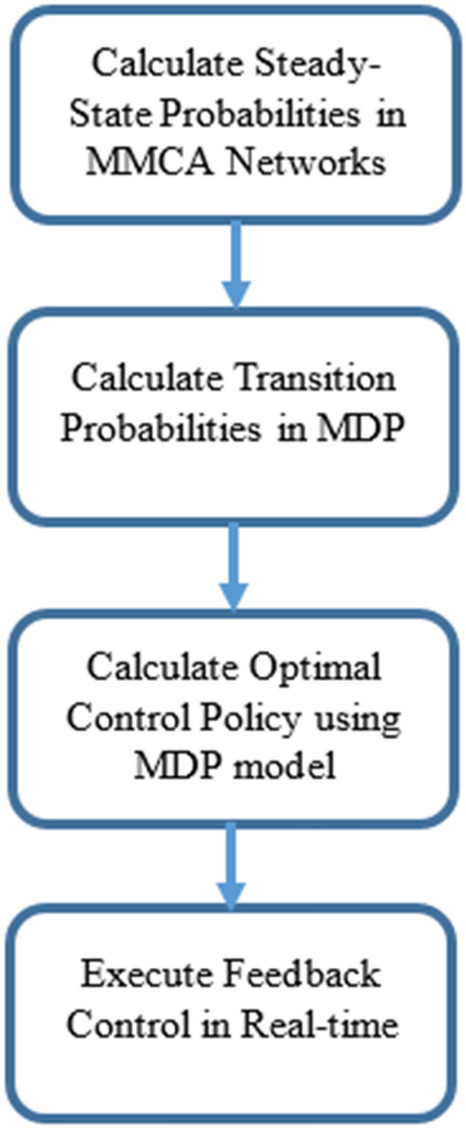
Implementation strategy for multi-layered approach

Major steps involved in the implementation of the proposed approach are shown in Fig. 4. As shown in the figure, first, we need to calculate the steady state probabilities corresponding to each individual in each network using the equations in (4). Then these steady state values are used to calculate the intermediate values in (12), (13), (17), and (18) that are used for calculation of transition probabilities in the MDP model.

Next step is to calculate the MDP policy using any of the standard stochastic dynamic programming algorithms such as value iterations or policy iterations. Execution of the policy in real time shall be achieved by feeding the data pertaining to each network, i.e., fraction of infected individuals and fraction of aware individuals to the MDP and resulting optimal decision is obtained from the optimal decision policy.

### B. Calculation of optimal control policy

Calculation of the optimal policy for infinite time horizons using the value iteration method is discussed. This method uses the concept of the value function in order to determine the optimal policy in an iterative manner. The value of a state ($V\left(x\right):X\to {\mathbb{R}}$) is a measure of importance of a state in terms of how less it costs and how likely it is to lead to another state with low cost using appropriate decision. Value iteration method uses arbitrarily assigned initial values of the states along with the transition probabilities in the MDP model to determine the optimal value of each state (${V}^{*}\left(x\right)$). Based on these optimal values, optimal decisions are calculated for each state. An optimal decision ${d}^{*}\in D$ as a function of state $x\in X$ is known as optimal policy (${\Pi }^{*}\left(x\right):X\to D$). Since the method is applied to stochastic decision-making problems, the optimization is in terms of the expected value given as

22 $$\underset{\Pi }{\mathrm{min}}E\left[\sum_{h=0}^{\infty }{\eta }^{h}J\left(x\left(h\right),d\left(h\right)\right)|\Pi \right]$$

Note that the expected value function ($E\left[.\right]$) in (22) is conditioned upon the control policy $\Pi$. The expected value of a random variable is an average of the probable values. In our case, the cost in a sequence of decision-making horizon $h$ is random because we cannot determine the exact sequence of states that will result from making decisions under the policy $\Pi$. The decision-making horizon *h* in a value iteration is assumed to be infinite. Also, $\eta$ is the discount factor as discussed in Sect. 2.2. The value of each state is calculated as

23 $$ V\left( x \right) = _{d \in D}^{max} \left( { - J\left( {x,d} \right) + \eta \mathop \sum \limits_{x^{\prime} \in X} F\left( {x^{\prime},d,x} \right)V\left( {x^{\prime}} \right)} \right) $$

From (23), the value of a state depends upon the sum of cost incurred by that state and the value of the states that it can lead to. In the value iteration algorithm, we initially assign arbitrary values to the states and then update the values iteratively using the following relation.

24 $$ V_{i + 1} \left( x \right) \leftarrow _{d \in D}^{max} \left( { - J\left( {x,d} \right) + \eta \mathop \sum \limits_{x^{\prime} \in X} F\left( {x^{\prime},d,x} \right)V_{i} \left( {x^{\prime}} \right)} \right) $$

Note that the iteration in (24) can be started with arbitrary (finite) state values and after sufficient number of iterations, the value function converges to an optimal value for each state (${V}^{*}\left(x\right)$). Once the optimal value is obtained, the optimal policy is calculated using the following expression

25 $$ {\Pi }^{*} \left( x \right) = \mathop {{\text{argmax}}}\limits_{d \in D} \left( { - J\left( {d,x} \right) + \mathop \sum \limits_{x^{\prime} \in X} F\left( {x^{\prime},d,x} \right)V^{*} \left( {x^{\prime}} \right)} \right) $$

Here V* represents optimal value and ${\Pi }^{*}\left(x\right)$ represents optimal policy or optimal policy function that provides an optimal decision ${d}^{*}\in D$ for each state $x\in X$. Note that the optimal policy in this case is stationary, i.e., the optimal decision at each state is independent of the time at which the state is reached.

### Relationship among the networks

The relationship among the networks is represented using the graph theory where each network is a node and the link between the two networks is an edge. A graph $\left(\mathcal{N},\mathcal{E}\right)$ where $\mathcal{N}$ is a set of nodes and $\mathcal{E}$ is a set of edges is characterized by an adjacency matrix. Adjacency matrix is a square matrix of the size equal to the number of networks in the model. Each element ($i,j$) of an adjacency matrix is either 1 (meaning that network $i$ is connected with network $j$) or 0 (meaning that network $i$ has no connection with network $j$, i.e., the individuals in both networks do not interact or communicate). The impact of interconnection among the networks (as far as the centralized control MDP is concerned) is upon the calculation of the transition probabilities. There are two possible approaches. One approach is to modify the transition probability Eqs. (9–18) in order to incorporate the impact of interaction among the networks upon the probability of change in awareness ratio and the infection ratio. A second approach to deal with the interconnected networks (that has been adopted in this paper) is to combine all interconnected networks into a single network. In this way, the interaction in incorporated in the consolidated adjacency matrices of the contact layer and the communication layer within the combined network (which is treated as a single network).

Now, one may argue that if the networks are independent, why centralized control (rather than the distributed control). While in principle, the distributed control is possible, e.g., having a separate MDP based policy for each network. However, in real life situations, the budget is allocated to a town or a city involving various sub-communities, i.e., shopping malls, cinemas, offices, schools, banks etc. Since the MDP policy decides on the allocation of resources, it is more realistic to have one MDP for multiple networks that lie within the scope of one decision making authority, e.g., a county or a municipal office etc.

### Computational complexity analysis

Discussion regarding the computational complexity is important wherever an MDP-based solution is involved in a stochastic optimization problem. In the problem at hand in this paper, there are two sources of complexity, one is the total number of networks ($M$) and the other is the precision with which the infection ratio and the awareness ratio are being measured ($\Delta$). Consequently, the size of the state space in MDP is given by

26 $$\left|X\right|={\left(\frac{\left(1+\Delta \right)}{\Delta }\right)}^{2M}$$

**Fig. 5:**
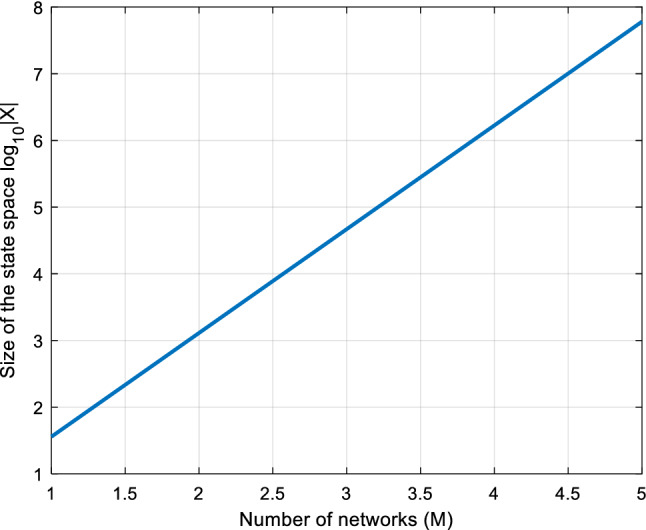
Size of the state space with respect to the number of networks where ${\varvec{M}}=3$ and ${\varvec{\Delta}}=0.2$

The above complexity is calculated from Eq. (7) based on the fact that there are $2M$ variables in each state and each of the $2M$ variables has $\left(\frac{1}{\Delta }+1\right)$ possible values. In order to have a rough estimate regarding the implication of (27), consider $M=3$ and $\Delta =0.2$. This implies a total of 46,656 states. This number grows exponentially with $M$ as shown in Fig. 5. Notice that the computational complexity involved in the calculation of the optimal policy from the MDP model is proportional to the square of the size of the state space. With current computing capabilities (gigahertz processors), the complexity of the order of ${10}^{9}$ calculations is affordable which may translate somewhere between 5 and 7 networks depending upon the value of $\Delta$. Therefore, a real life application of the proposed model, does demand distributed control in a sense that small blocks of a town may be isolated via something like a *smart lockdown* (which has been practiced by many governments during the COVID-19 pandemic). Smart lockdown limits the number of networks (houses and other buildings) to be dealt with by a single decision making authority. In terms of hospitals, the reduction in the complexity may be achieved by allocating various blocks within a hospital to the individuals within a sub-group of networks that is being controlled with a single MDP-based policy.

## Numerical example

In this section, a numerical example with three MMCA based networks governed by an MDP has been discussed. This numerical example not only provides insight about how to implement the proposed approach, but it also serves as a guideline for evaluation of the resulting optimal policy.

Note that the numerical example presented in this section consists of only three networks where each network has three nodes. The reason for presenting small network is to facilitate the understanding of the parameter values and the calculations involved in the model. For networks with more than three nodes, the size of the connectivity matrices (A and B) would be larger. Similarly, the steady state probability values (presented later in Table 1) would be large in number. For example, for a three-node network, we need 15 values per network (a total of 45 values for three networks as shown in Table 1). But if we were to present an example of five nodes, there would have been 75 steady state values for three networks that would be cumbersome to add in a Table. On the other hand, the three-node network is able to exhibit enough structure in the problem that is needed for understanding the implications and trends in the optimal control policy.

**Table 1:** Steady state probability values for MMCA networks

Network 1	Network 2	Network 3
${p}_{1}^{US}=0.0174,$	${p}_{1}^{US}=0.0110,$	${p}_{1}^{US}=0.0968,$
${p}_{2}^{US}=0.066,$	${p}_{2}^{US}=0.0110,$	${p}_{2}^{US}=0.0968,$
${p}_{3}^{US}=0.011,$	${p}_{3}^{US}=0.0464,$	${p}_{3}^{US}=0.0968,$
${p}_{1}^{AS}=0.1559,$	${p}_{1}^{AS}=0.1538,$	${p}_{1}^{AS}=0.2654,$
${p}_{2}^{AS}=0.3019,$	${p}_{2}^{AS}=0.1538,$	${p}_{2}^{AS}=0.2654,$
${p}_{3}^{AS}=0.1538,$	${p}_{3}^{AS}=0.3256,$	${p}_{3}^{AS}=0.2654,$
${p}_{1}^{UI}=1.56\times {10}^{-4},$	${p}_{1}^{UI}=1\times {10}^{-4},$	${p}_{1}^{UI}=4.2\times {10}^{-4},$
${p}_{2}^{UI}=2.87\times {10}^{-4},$	${p}_{2}^{UI}=1\times {10}^{-4},$	${p}_{2}^{UI}=4.2\times {10}^{-4},$
${p}_{3}^{UI}=1\times {10}^{-4},$	${p}_{3}^{UI}=2.031\times {10}^{-4},$	${p}_{3}^{UI}=4.2\times {10}^{-4},$
${p}_{1}^{AI}=0.0114,$	${p}_{1}^{AI}=0.0105,$	${p}_{1}^{AI}=0.0133,$
${p}_{2}^{AI}=0.0126,$	${p}_{2}^{AI}=0.0105,$	${p}_{2}^{AI}=0.0133,$
${p}_{3}^{AI}=0.0105,$	${p}_{3}^{AI}=0.0122,$	${p}_{3}^{AI}=0.0133,$
${p}_{1}^{UR}=0.0768,$	${p}_{1}^{UR}=0.0602,$	${p}_{1}^{UR}=0.0649,$
${p}_{2}^{UR}=0.0511,$	${p}_{2}^{UR}=0.0602,$	${p}_{2}^{UR}=0.0649,$
${p}_{3}^{UR}=0.0602,$	${p}_{3}^{UR}=0.0415,$	${p}_{3}^{UR}=0.0649,$
${p}_{1}^{AR}=0.7384,$	${p}_{1}^{AR}=0.7643,$	${p}_{1}^{AR}=0.5592,$
${p}_{2}^{AR}=0.5681,$	${p}_{2}^{AR}=0.7643,$	${p}_{2}^{AR}=0.5592,$
${p}_{3}^{AR}=0.7643.$	${p}_{3}^{AR}=0.5741.$	${p}_{3}^{AR}=0.5592.$

### Parameter values

**Fig. 6:**
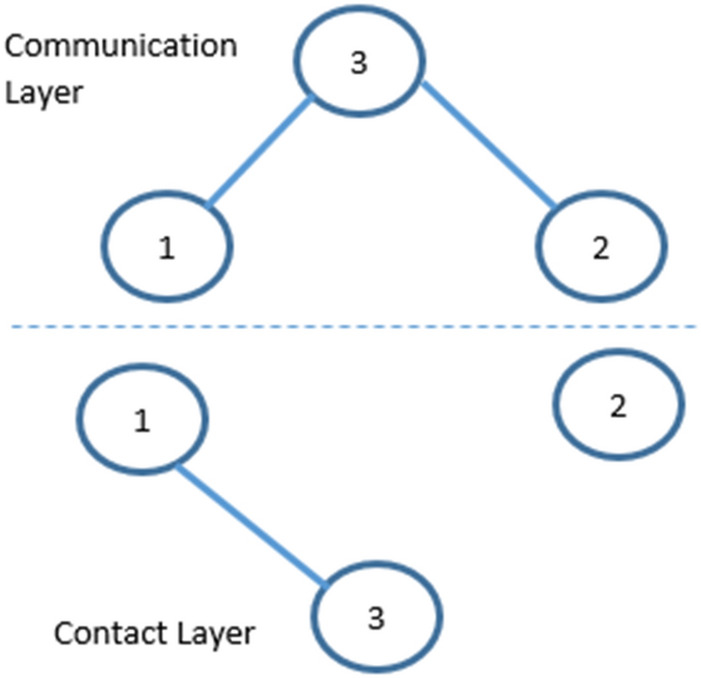
Topology of network 1

The example discussed considers three networks ($M=3$) and each network consists of three individuals ($m=3$). Network 1 is assumed to be consisting of individuals that have a chain-type communication where some of the individuals do not communicate directly (see Fig. 6). Connectivity within network 1 is given by

27 $$A1 = \left[ {\begin{array}{*{20}c}    1 & {amp;0} & {amp;1}  \\    0 & {amp;1} & {amp;1}  \\    1 & {amp;1} & {amp;1}  \\   \end{array} } \right],\,B1 = \left[ {\begin{array}{*{20}c}    1 & {amp;0} & {amp;1}  \\    0 & {amp;1} & {amp;0}  \\    1 & {amp;0} & {amp;1}  \\   \end{array} } \right]$$

Here, A1 represents a connectivity matrix for the communication layer of the network whereas B1 represents the connectivity layer of the network. Corresponding graphical illustration of network topology is presented in Fig. 6. Network 2 is selected to have direct communication links among all three members. Consequently, network 2 is set up with the following connectivity matrices

28 $$A2 = \left[ {\begin{array}{*{20}c}    1 & {amp;1} & {amp;1}  \\    1 & {amp;1} & {amp;1}  \\    1 & {amp;1} & {amp;1}  \\   \end{array} } \right],\,B2 = \left[ {\begin{array}{*{20}c}    1 & {amp;1} & {amp;0}  \\    1 & {amp;1} & {amp;0}  \\    0 & {amp;0} & {amp;1}  \\   \end{array} } \right]$$

For the third network, we assume no communication among the members, i.e., the individuals are completely isolated, therefore, $A3=B3={I}_{3\times 3}$. The selection of three different networks with less, moderate, and high level of communication ensures diversity in the numerical example.

The parameter values have been selected based on the discussions in [11] and some adaptations have been made in order to create a difficult situation. For example, the probability of getting infected for the aware individual has been selected to be 0.1 instead of 0. Also, the probability of becoming aware has been selected based on the proportion of the educated versus uneducated people in Pakistan. Curing rate has been selected to be lower than what has been observed for COVID recently (to make the problem more challenging). Consequently, the values of the parameters are given as follows

29 $$ \begin{gathered} \lambda = 0.2,\beta^{A} = 0.1,\beta^{U} = 0.2,p_{i}^{A} \left( 0 \right) = 0.2,\delta = 0.01, \hfill \\ \mu = 0.7,\nu = 0.9 \hfill \\ \end{gathered} $$

Based on the above-mentioned parameter values, the steady state probabilities for all three networks were computed using (4) which turned out to be as given in Table 1.

Based on above mentioned steady state probabilities, the intermediate probability values for MDP are as follows

30 $$ \begin{gathered} \overline{{p_{1}^{A} }} = 0.9056,\overline{{p_{2}^{A} }} = 0.9231,\overline{{p_{3}^{A} }} = 0.8379 \hfill \\ \overline{{p_{1}^{U} }} = 0.0944,\overline{{p_{2}^{U} }} = 0.0769,\overline{{p_{3}^{U} }} = 0.1621 \hfill \\ \overline{{p_{1}^{I} }} = 0.0117,\overline{{p_{2}^{I} }} = 0.0112,\overline{{p_{3}^{I} }} = 0.0137 \hfill \\ \overline{{p_{1}^{A} }} = 0.7530,\overline{{p_{2}^{A} }} = 0.7549,\overline{{p_{3}^{R} }} = 0.6241 \hfill \\ \end{gathered} $$

Next, the optimal control policy for the resulting MDP model is calculated using value iteration with $\eta =0.9$. Salient features of the calculated policy are discussed in the next subsection.

### Optimal control policy

For the calculation of the optimal policy, the cost of treatment drive and awareness campaign is set to be ${\alpha }_{3}=10,{\alpha }_{4}=100$ and the cost weighting on infection ratio and awareness ratio is set as ${\alpha }_{1}=10,{\alpha }_{2}=100$. Priorities of the networks have been set as ${k}_{1}=2,{k}_{2}=3,{k}_{3}=1$. Note that network 3 (that is hospital) has lowest priority because it already is network where there is lesser need for additional awareness campaign.

**Fig. 7:**
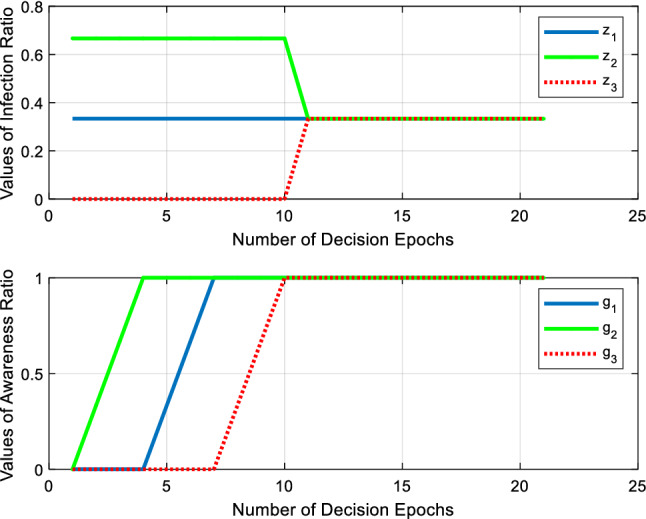
Sample trajectory with optimal control policy where ${{\varvec{z}}}_{{\varvec{i}}}$ is the infection ratio for network ${\varvec{i}}$ and ${{\varvec{g}}}_{{\varvec{i}}}$ is the corresponding awareness ratio

A sample trajectory with optimal control policy is presented in Fig. 7. Note that the initial conditions for this trajectory are such that all three networks have zero awareness and the infection ratio is 0.33, 0.66, and 0 respectively in network 1, network 2, and network 3. It can be noted from the bottom graph in Fig. 7 that the first priority of the optimal policy is to execute awareness campaigns in all three networks resulting in full awareness among the networks (this behavior is reflective of the high cost set against the lack of awareness ${\alpha }_{2}$). Top graph indicates that (after the awareness has been maximized) the infected individuals from network 2 (which is the highest priority network) have been transferred to the hospital (network 3). There are two characteristics of the optimal policy worth discussing here. One is that the decision making in the optimal policy is sequential, i.e., the awareness campaign and the treatment drive are not launched simultaneously. In real life scenarios, the decisions could be made simultaneously, in order to model simultaneous decision making in the MDP, we must define a single decision as a combination of multiple decisions, i.e., we may define a decision ${d}_{T1,A2}$ meaning that treatment drive is being executed for network 1 and the awareness campaign is being executed in network 2. Such definition of decisions results in increase in the decision space as all possible combinations of treatment drives and awareness campaigns are to be included. Another (simpler) way of dealing with sequential decision making is to have a smaller decision epoch (with same decision space as define in (8)). A decision epoch is time interval between the two consecutive decisions. A smaller epoch results in faster decision making. However, a drawback of having smaller epoch is that the value of the next state could virtually be the same. In our case, no change in infection ratio may be observed during the time interval between the two decisions (as it takes time for an individual to recover from the disease). Second characteristic of the optimal policy worth discussing is the prioritization among the networks. For example, in the results shown in Fig. 7, the optimal policy does not launch any treatment drive in network 1. Such behavior of prioritization is important when the resources are limited (note that in our example, the cost of treatment campaign is ten times that of the awareness campaign). Another justification for this behavior is that the infection ratio in Network 1 (0.33) is within the threshold of epidemic outbreak (0.35 for $\gamma =1$, see Eq. 6). Note that the epidemic threshold for Network 1 (for $\gamma =1$) is $\mu$ over the largest eigen value of $B1$ matrix in Eq. (27). Although the MDP does not consider this threshold explicitly, it is reflected in the policy because we utilize the steady state probability values from the two-layer networks into the state transition model of the third MDP layer.

**Fig. 8:**
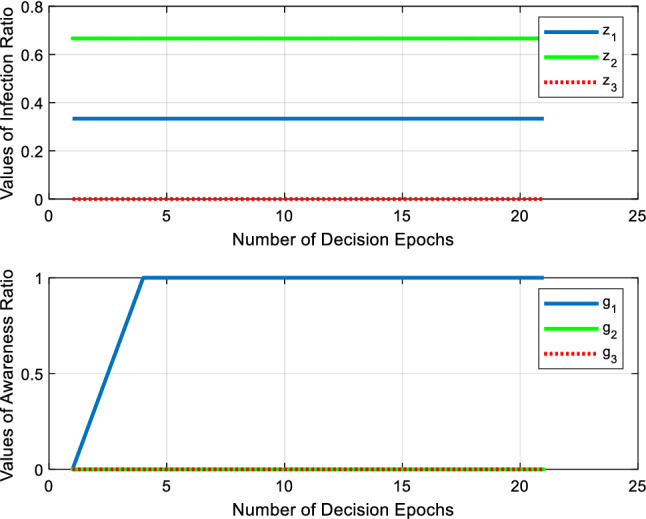
Sample Trajectory without optimal control policy where ${{\varvec{z}}}_{{\varvec{i}}}$ is the infection ratio for network ${\varvec{i}}$ and ${{\varvec{g}}}_{{\varvec{i}}}$ is the corresponding awareness ratio

For comparison of the results from the optimal policy, we also present the results of sample trajectories without the optimal policy (generated based on maximum likely state transitions) in Fig. 8. Notice that due to low recovery and infection rates chosen in our example, the infection does not change in any of the networks (see Eq. (30)). Also, the trajectories only indicate the maximum likely sequence. In general, the disease will eventually spread in the infection ratio is above the threshold presented in Eq. (6). On the other hand, due to strong connectivity in the Network 1, the awareness about the disease spreads without any awareness campaign. Another factor for the spread of awareness in any network is the initial awareness in some of the nodes of the network. In general, the infection ratio may increase or decrease even without any awareness or treatment campaigns depending upon the rate of the spread of infection.

Another comparison between the presence and absence of the optimal policy in terms of the cost incurred (as per Eq. (20)) is presented in Table 2. The comparison presents the cumulative cost incurred with and without the optimal policy for five sample trajectories. The length of each trajectory is 20 decision epochs (recall that the decision epoch is a user specified time duration between the two consecutive policy decisions). It is evident form the results that in general, the cost without the policy may be as much as three times of that with the optimal policy. However, this difference is highly dependent upon the initial condition. Specifically, for the initial conditions involving high level of awareness ratio, the both the cost magnitude and the difference between the two costs are low. In extreme case, with full awareness across all three networks and zero initial infection ratio, the cost with the optimal policy is equal to that without the optimal policy (both cost values are zero).

**Table 2:** comparison of cost (equ. 20) with sample trajectories of length 20 decision epochs

Sr. No	Initial state ${x}_{0}=\left[{z}_{\mathrm{1,0}},{z}_{\mathrm{2,0}},{z}_{\mathrm{3,0}},{g}_{\mathrm{1,0}},{g}_{\mathrm{2,0}},{g}_{\mathrm{3,0}}\right]$	Cost with the optimal policy	Cost without the optimal policy
1	${x}_{0}=\left[\mathrm{0.33,0.66,0},\mathrm{0,0},0\right]$	8600	26,800
2	${x}_{0}=\left[\mathrm{0,0},\mathrm{0,0},\mathrm{0,0}\right]$	7200	25,200
3	${x}_{0}=\left[\mathrm{1,0.66,0.33,0.66,1},0.33\right]$	3300	6,800
4	${x}_{0}=\left[\mathrm{1,1},\mathrm{1,0},\mathrm{0,0}\right]$	10,800	28,800
5	${x}_{0}=\left[\mathrm{0,0},\mathrm{0,1},\mathrm{1,1}\right]$	0	0

**Fig. 9:**
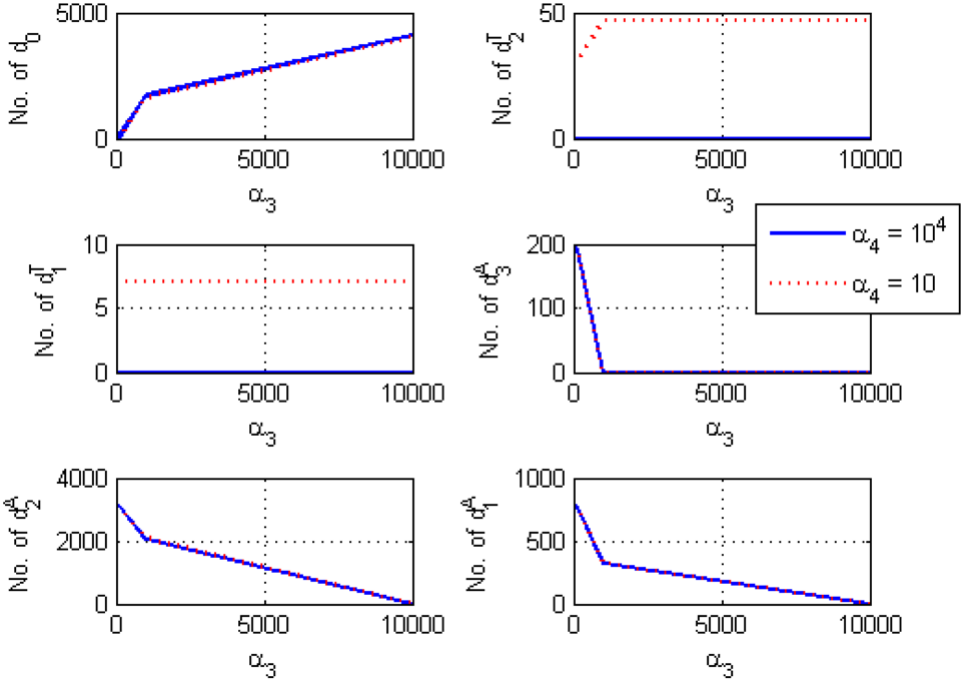
Trends in optimal policy with respect to ${\boldsymbol{\alpha }}_{3}$. Specifically, the frequency of each decision, e.g., no operation (${{\varvec{d}}}_{0}$), treatment campaign in network 1 (${{\varvec{d}}}_{1}^{{\varvec{T}}}$), awareness campaign in network 2 (${{\varvec{d}}}_{2}^{{\varvec{A}}}$) etc. is plotted against the ranges of the parameter (${\boldsymbol{\alpha }}_{3}$) values. Furthermore, the blue graphs correspond to the value ${\boldsymbol{\alpha }}_{4}={10}^{4}$ and the doted red graphs correspond to ${\boldsymbol{\alpha }}_{4}=10$

**Fig. 10:**
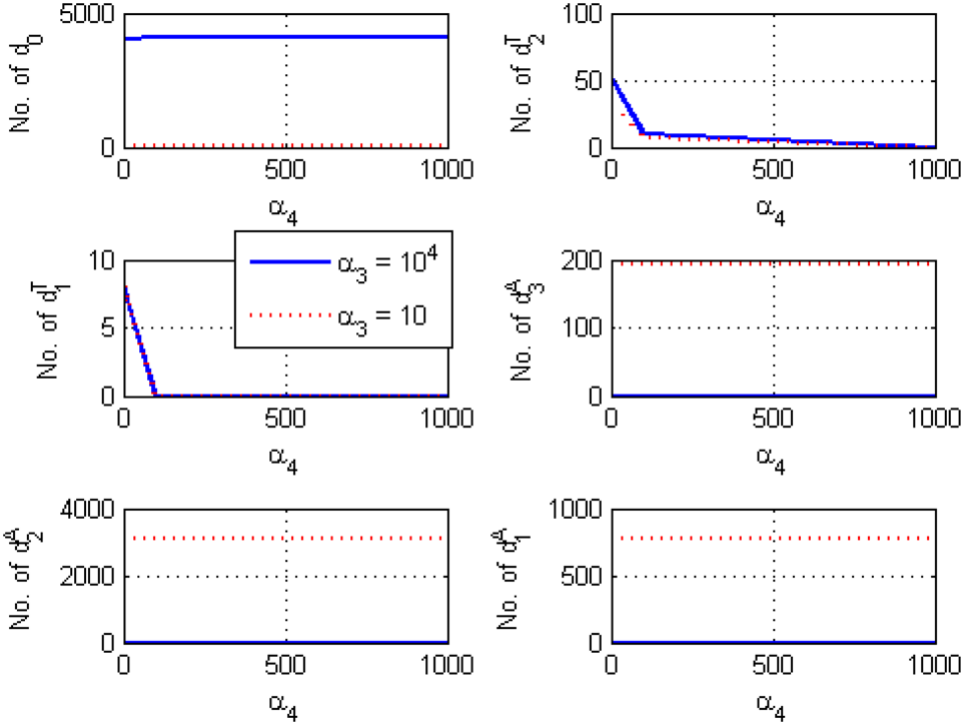
Trends in optimal policy with respect to ${\alpha }_{4}$. Specifically, the frequency of each decision, e.g., no operation (${d}_{0}$), treatment campaign in network 1 (${d}_{1}^{T}$), awareness campaign in network 2 (${d}_{2}^{A}$) etc. is plotted against the ranges of the parameter (${\alpha }_{4}$) values. Furthermore, the blue graphs correspond to the value ${\alpha }_{4}={10}^{4}$ and the doted red graphs correspond to ${\alpha }_{4}=10$

In order to develop further insights into the trends in the optimal control policy, the frequency of each decision has been plotted with respect to cost parameter ${\alpha }_{3}$ and ${\alpha }_{4}$ in Fig. 9 and Fig. 10 respectively. In both of the figures, all plots have number of occurrences of a decision in the optimal policy as y-axis value and on the x-axis are the corresponding parameter values used for calculating the optimal policy. There are three types of decisions as discussed earlier in Sect. 2.2., i.e., ${d}_{j}^{T}$ means launching treatment campaign in network $j$, ${d}_{0}$ means no-action, and ${d}_{j}^{A}$ means launching awareness campaign in network $j$. For each value-pair of the parameters (${\alpha }_{3}$ and ${\alpha }_{4}$), the optimal policy has been calculated and the histogram (number of occurrences) of decisions in the optimal policies have been plotted against the corresponding parameter values. It is clear in Fig. 9 that the number of occurrences of awareness campaigns reduces as ${\alpha }_{3}$ increases (recall that ${\alpha }_{3}$ is the cost of an awareness campaign) whereas the number of occurrences of treatment decisions remains almost the same except for one case where the frequency increases (dotted graph on top right of Fig. 9).

Trends in Fig. 9 indicate that increase in ${\alpha }_{4}$ results in the decrease in the frequency of treatment decisions (recall that ${\alpha }_{4}$ is the cost of a treatment campaign) in the optimal control policy. Such results can be used by decision making officials for devising optimal response to changing circumstances and also for prediction of funds required in order to optimally control the spread of an epidemic disease. Furthermore, the increase in ${d}_{0}$ indicates that, as the resources become scarce or expensive, the optimal decision is to refrain from spending the same without justification. Main advantage of the analysis of the optimal policy with respect to cost is the determination of the thresholds where the emergency situation may be declared. Similar, analysis may be performed with respect to changes in the probabilities, for example, the curing rate $\mu$.

### Simulating networks with more nodes

The above example has discussed three networks each having three nodes. While we have already discussed the impact of having increase in the number of networks on the computational complexity of an MDP layer, we still need to discuss the impact of having more nodes within each network. For example, what would happen if each of the three networks discussed in the above example had 100 nodes instead of three? The good news is, that the complexity of the MDP layer (i.e., the number of states in the MDP) would still be the same regardless of the number of nodes in each network. On the other hand, increase in the number of nodes would result in the changes in size of the A, B matrices given in the Eqs. (27) and (28). For a hundred node network, the size of A and B matrices would be $100\times 100$. This is not a computational issue since we do not need to calculate the inverse of any of these matrices. We would use Eqs. (1–4) in order to calculate the steady state values of the probabilities associated with the network layers. Finally, the corresponding probabilities for the MDP would be computed using Eqs. (12), (13), (17), and (18). The results would be similar to the values presented in the Eq. (30). Once we have the values for the MDP-layer transition probabilities, it does not matter how many nodes each network has. This is because all of the information regarding the connectivity in the network layers has been consolidated into probabilities regarding the spread of information and the spread of the disease. Having said that, it would still be an avenue of future research to study the impact of various connectivity patterns on the steady state probabilities in the MDP layer. In the opinion of the author, these probabilities would depend more on how strongly or weakly the network is connected and depend less on the size (number of nodes) of each network. Still a concise investigation on this issue is an avenue of future research.

## Conclusion

This paper has discussed a multi-layered model for epidemics control that facilitates calculation of optimal control policy. The proposed model is an extension of an existing multiplex network approach for modeling the epidemic spread. Implementation of the proposed approach involves calculation of steady state probabilities for social networks and incorporation of the same into transition probabilities in an MDP model. The resulting optimal policy has been discussed through a numerical example and some trends in the optimal policy have been highlighted. It is evident from the numerical results that the optimal policy finds a tradeoff between the resource consumption and the corrective measures. For example, the trends indicated in previous section show that the treatment and the awareness campaign frequency is not solely dependent upon the infection ratio, rather it also considers the cost of treatment and the cost of spreading awareness (Fig. 9 and Fig. 10). This implies that the infection ratio can be minimized only if we have enough resources for spreading the awareness (i.e., if the cost of awareness campaign is low). Also, the treatment campaigns depend upon the cost of the treatment which in turn depends upon the availability of hospitals and medical staff. Further investigation into the structure of the optimal policy and development of theoretical results is an avenue of future research on this topic. Major takeaways from the current research are, (1) MDP policy makes sequential decisions, therefore, the decision epoch may be defined with careful deliberations while implementing the proposed approach, (2) The computational complexity grows quickly as the number of networks increases, therefore, a distributed (multiple MDP-based) approach is recommended for large scale problems. Such an approach may require smart lockdowns by the authorities (3) A spinoff benefit of the proposed approach is that the model allows for cost and chance analysis that can help the authorities to define thresholds of emergencies in terms of the availability of resources and the curing rate.

Based on the proposed model and the results discussed in the paper, it is evident that the proposed model can be used for the government for taking preventive measures against the spread of an epidemic disease in the following manner. First, the population shall be divided into social and contact networks, e.g., the people who communicate frequently with each other and the people who live together or work together such as family members and coworkers. Next, the cost of hospitalization, vaccination, and the cost of awareness campaign shall be added appropriately in the proposed model for the calculation of the optimal policy. The policy provides a guideline (optimal decision) as a function of existing situation (system state). One future direction is to modify the proposed approach by incorporating available resources such as health budget. Such modification may enable the resulting policy to provide guidelines for different amounts of health budgets. In this way, one can generate appropriate estimation of health budgets based on the statistical data regarding the spread of an epidemic disease. Note that the proposed model is only effective when the statistical data about the spread of a disease (or an appropriate approximation) is available for the calculation of associated probabilities.

Although the proposed method presents an improvement on the existing networked control approaches, still there are some key challenges and limitations involved that should be discussed here. Two major limitations of the proposed approach are scalability and availability of information. In terms of scalability, decentralization is inevitable. For large population groups, it may not be feasible to have a single MDP for making decisions. Hence the problem has to be decomposed into smaller subproblems. Second challenge regarding the availability of information is also critical because the state information is required for the determination of control policy (state information includes the exact ratio of infection and awareness among the population). Furthermore, the state transition probabilities are also determined from the statistical data that must be collected before the practical implementation of the proposed approach. In the absence of correct information or data, the MDP model should be replaced with a Partially Observable Markov Decision Process (POMDP) model. Regarding the lack of statistics, machine learning may be employed for learning the trends in state transitions. Such investigations constitute the avenues for future work. Another possible future direction is to incorporate the study on human behavior (as discussed in [17]) into the network layer model in the proposed approach (i.e., feeding the state transition probabilities in the MDP by incorporating the cumulative disease information in the MRFC model). Such an innovation may reveal useful insights regarding the effective control of the spread of epidemics.

## Data Availability

Data sharing not applicable to this article as no datasets were generated or analyzed during the current study.
